# α-Tocopherol influences glycaemic control and *miR-9-3* DNA methylation in overweight and obese women under an energy-restricted diet: a randomized, double-blind, exploratory, controlled clinical trial

**DOI:** 10.1186/s12986-018-0286-7

**Published:** 2018-07-11

**Authors:** Rafaella Cristhine Pordeus Luna, Mayara Karla dos Santos Nunes, Mussara Gomes Cavalcante Alves Monteiro, Cássia Surama Oliveira da Silva, Rayner Anderson Ferreira do Nascimento, Raquel Patrícia Ataíde Lima, Flávia Cristina Fernandes Pimenta, Naila Francis Paulo de Oliveira, Darlene Camati Persuhn, Aléssio Tony Cavalcanti de Almeida, Alcides da Silva Diniz, Cristina Wide Pissetti, Rodrigo Pinheiro Toledo Vianna, Flavia Emília Leite de Lima Ferreira, Maria da Conceição Rodrigues Gonçalves, Maria José de Carvalho Costa

**Affiliations:** 1 Postgraduate in Nutrition Sciences, Health Sciences Center, Federal University of Paraíba (Universidade Federal da Paraíba), João Pessoa, Paraíba 58051-900 Brazil; 2 Postgraduate Program in Cellular and Molecular Biology, Federal University of Paraíba (Universidade Federal da Paraíba), João Pessoa, Paraíba 58059-900 Brazil; 3 Health and Nutrition Studies Interdisciplinary Center, Health Sciences Center, Federal University of Paraíba (Universidade Federal da Paraíba), João Pessoa, Paraíba 58051-900 Brazil; 4 Department of Internal Medicine, Health Sciences Center, Federal University of Paraíba (Universidade Federal da Paraíba), João Pessoa, Paraíba 58051-900 Brazil; 5 Departament of Molecular Biology, Federal University of Paraíba (Universidade Federal da Paraíba), João Pessoa, 58059-900 Paraíba Brasil; 6 Department of Economics, Postgraduate Program in Applied Economics and Economics of the Public Sector, Center for Applied Social Sciences, Federal University of Paraíba (Universidade Federal da Paraíba), João Pessoa, Paraíba 58059-900 Brazil; 7 Department of Nutrition, Graduate Program in Nutrition, Health Sciences Center, Federal University of Pernambuco, Recife, Pernambuco 50670901 Brazil; 8 Department of Obstetrics and Gynecology, Health Sciences Center, Federal University of Paraíba (Universidade Federal da Paraíba), João Pessoa, Paraíba 58051-900 Brazil; 9 Department of Nutrition, Graduate Program in Nutrition Sciences, Health Sciences Center, Federal University of Paraíba (Universidade Federal da Paraíba), João Pessoa, Paraíba 58051-900 Brazil; 10 Postgraduate in Nutrition Sciences, Health Sciences Center, Health and Nutrition Studies Interdisciplinary Center (NIESN), Federal University of Paraíba (Universidade Federal da Paraíba), Castelo Branco, João Pessoa, Paraíba 58051-900 Brazil

**Keywords:** DNA methylation, miR-9, *miR-9-1*, *miR-9-3*, Haemoglobin A1C, Fasting plasma glucose, α-Tocopherol, Obesity, Diet

## Abstract

**Background:**

Excess weight is a strong risk factor for the development of dysglycaemia. It has been suggested that changes in the metabolism microRNAs, small non-coding RNAs that regulate gene expression, could precede late glycaemic changes. Vitamin E in turn may exert important functions in methylation and gene expression processes. This study aimed to determine the effect of α-tocopherol on glycaemic variables and *miR-9-1* and *miR-9-3* promoter DNA methylation in overweight women.

**Methods:**

A randomized, double-blind, exploratory, placebo-controlled study was conducted in overweight and obese adult women (*n* = 44) who ingested synthetic vitamin E (all-rac-α-tocopherol), natural source vitamin E (RRR-rac-α-tocopherol) or placebo capsules and were followed up for a period of 8 weeks. Supplemented groups also received dietary guidance for an energy-restricted diet. An additional group that received no supplementation and did not follow an energy-restricted diet was also followed up. The intervention effect was evaluated by DNA methylation levels (quantitative real-time PCR assay) and anthropometric and biochemical variables (fasting plasma glucose, haemoglobin A1C, insulin, and vitamin E).

**Results:**

Increased methylation levels of the *miR-9-3* promoter region (*P* < 0.001) and reduced haemoglobin A1C (*P* < 0.05) were observed in the natural source vitamin E group after intervention. Increased fasting plasma glucose was observed in the synthetic vitamin E group, despite the significant reduction of anthropometric variables compared to the other groups.

**Conclusions:**

α-Tocopherol from natural sources increased methylation levels of the *miR-9-3* promoter region and reduced haemoglobin A1C in overweight women following an energy-restricted diet. These results provide novel information about the influence of vitamin E on DNA methylation.

**Trial registration:**

ClinicalTrials.gov, NCT02922491. Registered 4 October, 2016.

**Electronic supplementary material:**

The online version of this article (10.1186/s12986-018-0286-7) contains supplementary material, which is available to authorized users.

## Background

Obesity is a pro-inflammatory state with a broad impact on health and is the main risk factor for metabolic diseases such as diabetes, with an alarming increase worldwide [1]. Epigenetic mechanisms such as DNA methylation and regulation by microRNAs (miRs) play a central role in the obesogenic environment [2, 3]. Conditions associated with obesity, such as chronic inflammation, hyperglycaemia and hyperlipidaemia, are related to changes in DNA methylation status and gene expression [2, 4]. MiRs in turn can affect several signal pathways with impact on chronic inflammation, apoptosis, and abnormal cell cycle progression [3].

Methylation of DNA in promoter regions, particularly in CpG dinucleotides (cytosine-phosphate-guanine), is more often associated with transcriptional repression, while lower levels of methylation are related to increased transcription [5–7], although this relationship is not universal [8]. MiRs are small RNAs of approximately 22 nucleotides, which, unlike messenger RNA (mRNA), do not encode protein. The miRs perform functions of mRNA repression or degradation, leading to the impediment of translation and acting as post-transcriptional regulators [9–11].

Transcription of miRs can also be epigenetically regulated by methylation in CpG islands [12, 13]. In the human genome, there are three different regions that can be transcribed in the same mature miR-9 sequence: miR-9-1 (chromosome 1), miR-9-2 (chromosome 5) and miR-9-3 (chromosome 15) [10, 14]. MiR-9 plays a role in insulin and glucose homeostasis, in vitro and in vivo, and its dysregulation has been associated with obesity, diabetes, and non-alcoholic fatty liver disease [14–17]. However, despite these findings, the impact of nutritional interventions related to obesity on miR methylation levels has not yet been described, with most studies investigating genes encoding mRNA [4].

α-Tocopherol is a signalling molecule involved in an extensive network, with effects on gene regulation, particularly by modulation of the activity of several signal transduction enzymes and transcription factors [18, 19]. In mice, tocopherols and tocotrienols reduced DNA damage and affected *DNA methyltransferase 1 (Dnmt1)* and *MutL homolog 1 (MLH1)* gene expression and methylation [20]. It was also demonstrated that differences in dietary vitamin E may affect hepatic miRs concentrations in rats; however little is known about potential regulatory effects of vitamin E on miRs [21].

Thus, for its possible influence on DNA methylation [20, 22, 23], vitamin E has been suggested as an epigenetically active nutrient as well as an important nutritional factor in preventing DNA damage caused by oxidative stress due to obesity [20]. In fact, obesity, associated with oxidative stress and inflammation, seems to increase the requirement for α-tocopherol [24, 25]. It has also been observed that approximately 79% of the world population has α-tocopherol blood levels below the levels at which health effects such as prevention of cardiovascular disease and different types of cancer may occur (< 30 μmol/L) [26]. According to a recent systematic review of α-Tocopherol global status, low dietary intake has also been reported, reaching values even below estimated average requirement for vitamin E in the US (EAR: 12 mg/day) [27]. Taken together, these observations highlight the potential of vitamin E on health [26, 28].

Two sources of α-Tocopherol, naturally sourced RRR α-Tocopherol (likely greatest effect on health outcomes) and synthetic all-racemic α-Tocopherol, are commonly consumed from foods and dietary supplements, respectively [29]. These different isomeric forms differ in bioactivity and human trials have been less conclusive than animal research about health beneficial effects of both sources a-Tocopherol [30]. It was demonstrated that vitamin E supplementation, with or without caloric restriction upregulated the mRNA expression of genes that encodes key enzymes involved in cholesterol metabolism, suggesting the maintenance of hepatic homeostasis in rats [31]. It is thought also that caloric restriction it might regulates many different metabolic pathways and attenuate the epigenetic changes occurring during the progress of aging [32]. Thus, this study aimed to determine the effect of α-Tocopherol (RRR α-Tocopherol and all-racemic α-Tocopherol) on glycaemic variables and *miR-9-1* and *miR-9-3* promoter DNA methylation in overweight and obese women under energy-restricted diet.

## Methods

### Subjects

Study subjects were recruited from the study titled “II Ciclo de Diagnóstico e Intervenção da Situação Alimentar, Nutricional e das Doenças não Transmissíveis mais Prevalentes da População do Município de João Pessoa (II DISANDNT/JP) [Cycle II of Diagnosis and Intervention of the Food, Nutritional and Non-Communicable Diseases Status of the Population of the Municipality of João Pessoa (II DISANDNT/JP)” [33, 34] which was conducted between May 2015 and May 2016. The study II DISANDNT/JP (technical advice: 0559/2013) and the clinical trial (technical advice: 1.697.641, CAEE 50410315.5.0000.5188) were approved by the Research Ethics Committee, Health Sciences Center, University of Paraíba, Brazil. The clinical trial was recorded on clinicaltrials.gov as NCT02922491. All subjects signed the informed consent before participating the study. The study was conducted in accordance with the Declaration of Helsinki.

A total of 166 overweight and obese (body mass index (BMI) ≥25 kg/m^2^) women were screened. We chose women because they represented a higher percentage and greater adherence to II DISANDNT/JP project [33, 34], thus, greater adherence likelihood to the clinical trial. We included women within the age range of 20–59 y with a preserved cognitive state. We excluded persons with a history of alcoholism, smoking, neuropsychiatric disorders, use of medication or medical diagnoses for chronic diseases with influence on the endocrine and metabolic system (diabetes, thyroid disorders, liver disease, kidney disease, cardiovascular disease and cancer), use of drugs known to interfere with folic acid metabolism (in the last 3 months), use of multivitamin or mineral supplements, use of anorexigenic or anabolic substances, pregnancy, plans to become pregnant, and loss of weight in the period prior to the study.

**Fig. 1:**
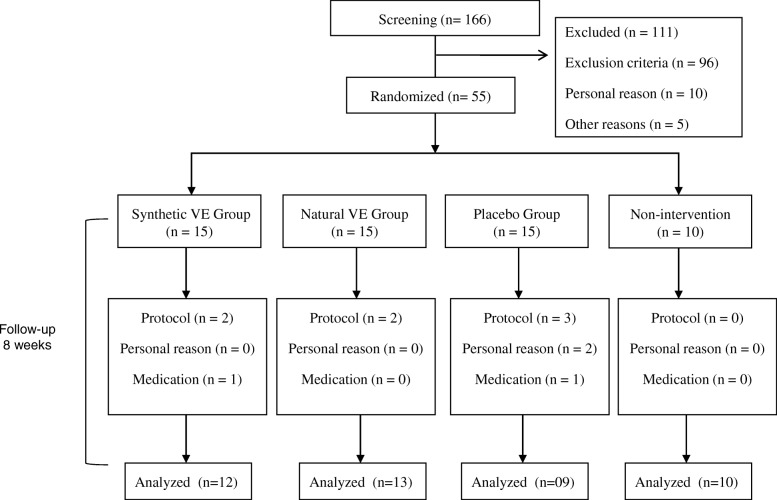
Flowchart of study subjects. VE: vitamin E. In total, 55 subjects were randomized in 4 groups being followed for a period of 8 weeks. The synthetic VE group received all-rac-α-tocopherol capsules, the natural VE group received RRR-α-tocopherol capsules, the placebo group received starch capsules. VE supplemented groups and placebo also received dietary guidance for energy-restricted diet. The non-intervention group did not receive any type of intervention during the 8-week period. Eleven subjects dropped out due protocol violation (*n* = 7), personal reason (*n* = 2), medication (*n* = 2). A total of 44 subjects were included in statistical evaluation. Before and after intervention were analyzed *miR-9-1* and *miR-9-3* methylation levels, anthropometric and biochemical variables (fasting plasma glucose, hemoglobin A1C, insulin, vitamin E)

After the initial screening, 55 subjects were block randomized and included in the trial. Fifteen women were allocated to each intervention group and 10 were allocated to the group that did not participate in any intervention through the randomization process using Stata® 13.0 software (College Station, Texas, USA). Eleven subjects did not complete the study. In total, 44 subjects completed the study and were included in data analysis (Fig. 1).

### Study design

The study was designed as a randomized, double-blind, exploratory, placebo-controlled study. One week before the start of vitamin E or placebo supplementation (plateau week), women received individual diet plans based on the dietary recommendations of the National Cholesterol Education Program (NCEP) [35] and the American Heart Association (AHA) [36]. The individual diet plans were performed using the food equivalent system proposed by Costa [37]. The energy content was based on the Estimated Energy Requirement (EER) to maintain the weight of women over 19 years of age with a BMI > 25 kg/m^2^ [38], subtracting approximately 500 to 1000 kcal, so that all consumed between 1200 and 1500 kcal to promote weight loss [39, 40]. We chose NCEP and AHA recommendations because the study’s women were at metabolic alterations risk, such as glycemic disorders. Thus, it is emphasized that in this intervention, women with glycemic dysregulation remained in the sample, except those already diagnosed with diabetes. We also implemented a weight loss diet due the genes evaluated were related to glucose metabolism in vivo (mice) in obesity [14, 41]. The recommended diet during the intervention consisted of about 55% carbohydrates, 30% fat (< 7% saturated fat), and 15% protein.

In the second week of the study, the women continued to follow the recommended individual dietary plan and started VE or placebo supplementation. The synthetic VE group received daily supplementation with 400 mg synthetic vitamin E (all-rac-α-tocopherol); the natural VE group received daily supplementation with 400 mg of natural source vitamin E from soybean oil (RRR-α-tocopherol); and placebo group 3 received daily supplementation with 400 mg of starch. The period of VE or placebo supplementation combined with energy-restricted diet lasted 8 weeks. Subjects met with a nutritionist every 15 days.

The subjects in the non-intervention group did not follow a diet plan, nor did they take vitamin E or placebo capsules; they were asked to continue their current eating habits over the 8-week period. At the end of the study, these participants received guidance for a similar energy-restricted diet to that received by the other groups.

Synthetic vitamin E capsules (all-rac-α-tocopherol acetate) were handled at the Roval LTDA (João Pessoa, Paraíba, Brazil), and natural vitamin E capsules (RRR-α-tocopherol), originating from soybean oil, were obtained from Carlson Lab (Arlington Heights, IL, USA) and had been used in a previous study with asthmatic patients [42]. Daily capsule intake was recommended during meals to enhance absorption. Synthetic vitamin E and placebo capsules had the same appearance and smell (opaque) and the natural capsules had a different appearance (gelatinous). Despite capsules appearance difference they were delivered individually to each participant’s home and the women did not meet during the study period. They did not know each other and live in different locations/neighborhoods keeping them blind during the study period. They remained blind for the ingestion of vitamin E or placebo capsules.

Regarding the quantity offered (400 mg), it is considered safe, according to the results of previous studies [43], and it is below the UL value for this vitamin, which is 1000 mg/day, with an Estimated Average Requirement (EAR) for adults of 12 mg/day, according to the Dietary Reference Intakes (DRIs) [44]. Therefore, we chose this dosage because no adverse effects were demonstrated so far and vitamin E supplementation may also help in the glycated haemoglobin control [43, 45]. Since results in mice may not be relevant to humans further studies are required to elucidate the potential beneficial or adverse effects of vitamin E [46]. Such studies will also help identify likely “nonresponders,” as well as those susceptible to untoward outcomes from supplementation with specific nutrients and their nutrient–gene interactions [47].

### Anthropometric and dietetic measurements

Weight and height were measured in triplicate, and the average of the three values was used. The BMI was then calculated as the body weight (kg) divided by the squared body height (meters), and the cut-off points recommended by the World Health Organization (WHO) were used [48]. Waist circumference (WC) was used to determine abdominal obesity [49] and waist-to-height ratio (WHtR) as an indicator of early health risk [50].

To evaluate the regular food intake of the individuals, four 24-h dietary recalls (24HR) were performed, two in the pre-intervention period, and two during the 4th and 8th intervention weeks. To complete the 24HR, a food photograph album with household measures was used. This food photograph was based on the actual weight of the average food intake validated for this population, thus minimizing possible biases of this method [51]. The foods were analyzed by the nutrition software Dietwin®, and the multiple source method (MSM) (https://msm.dife.de/) was used to estimate the regular intake of the individual from repeated measurements in a determined period.

### Biochemical measurements

The biochemical analyses were performed before and after the end of the nutritional intervention. Analyses of fasting plasma glucose by the enzymatic method [52], hemoglobin A1C (HbA1c) by high-performance liquid chromatography [53], insulin by electrochemiluminescence [54], HOMA-IR as fasting plasma glucose (mg/dL) x (fasting insulin (mg/dL) × 0.05551) / 22.5 [54] e vitamin E by ultra-performance chromatography [55] were performed. Blood samples were collected after overnight fasting in sterile vacuum tubes between 07:00 and 08:00 at patients’ homes, supervised by a nurse and the researcher responsible. Tubes for vitamin E analysis were carefully wrapped to avoid exposure to light. The samples were transported properly packed to the Clinical Pathology Laboratory HEMATO (João Pessoa, Paraíba, Brazil), where the analyses were conducted. The vitamin E analysis was carried out at the Hermes Pardini Laboratory (Minas Gerais, Brazil).

### DNA methylation analyses

The DNA from white blood cells were extracted using 10 mM TRIS-HCl, according to protocol described and adapted from Miller et al. [56]. Genomic DNA was modified by bisulfite from the Cells-to-CpG™ Bisulfite Conversion Kit (Applied biosystems, Life Technologies, California) according to the manufacturer’s instructions. The bisulfite conversion reaction was incubated at 65 °C for 30 min, 95 °C for 1.5 min, 65 °C for 30 min, 95 °C for 1.5 min, 65 °C for 30 min with a final incubation at 4 °C for up to 4 h in a PCR thermocycler. The analysis of methylation levels was performed by High Resolution Melting (HRM) Real Time PCR method in a Appplied Biosystems 7500 Fast System. PCR was performed in a total volume of 20 μl containing: 1× Buffer, 4 mM Mg + 2, 200 μM of each dNTPs (Qiagen), 250 nM of each primer, 5 μM SYTO® (Invitrogen), 1 U HotstarTaq DNA Polymerase (Qiagen) and 1 μl of bisulfite-modified DNA. The primers sequence were: miR-9-1-mF: 5′-GAT TTA GGT AGA GGT TTT TTT AgT TT-3′ (248 bp), miR-9-1-mR: 5′-TTA ACT ACC CAT TTC CCC TTT TAA T-3′, miR-9-3-mF: 5′-GTT TGT TTA TTT TTT TTG GTT TTT-3′ (220 bp), miR-9-3-mR: 5′-AAA TTA TAA AAA TCA TTT CTA CTT TC-3′ [57]**.** Primers were identified using UCSC website (http://genome.ucsc.edu/): miR-9-1, chr 1: 156390133–156,390,221; and miR-9-3, chr 15: 87,988,426–87,722,341 [57]. The PCR program consisted of an initial enzymatic activation at 94 °C for 10 minutos, followed by 35 ciclos de 94 °C/1 min, 60 °C/1 min and 72 °C/30 s, with a final extension at 72 °C for 10 min. The melting curves were normalized by calculation of the ‘line of best fit’ in between two normalization regions before and after the major fluorescence decrease representing the melting of the PCR product using the software provided with the HRM Software v2.0, provided by 7500 Fast System.

### Statistical analysis

Continuous variables were tested for normality and homogeneity of variances by the Shapiro-Wilk test and the Levene test, respectively. Variables with normal distribution were described as the means and standard deviation. The variables with non-Gaussian distribution were described as median and quartile interval and categorical variables, as proportions.

To evaluate possible pre- and post-intervention differences between *miR-9-1* and *miR-9-3* methylation levels in each group, we used the paired Wilcoxon test and boxplot graphs for representation. For the comparison of biochemical and dietary variables before and after the intervention in each group, Student’s paired t-test was used for glycaemic control variables and serum vitamin E, and the paired Wilcoxon test was used for dietary variables.

One-way ANOVA and post hoc Bonferroni test were used for comparisons among groups before and after intervention (glycaemic control variables and serum vitamin E), and the Kruskal-Wallis test and Mann-Whitney’s a posteriori test, for variables with non-normal distribution and/or heteroscedasticity (methylation levels and dietary variables). The χ2 test was used to compare categorical variables in the comparability among the pre-intervention groups. The statistical program used was Stata® 13.0 (College Station, Texas, USA). Statistical significance was determined as *p* < 0.05. It is noteworthy that in all statistical analyzes performed, the *p*-values adjusted by the Hommel procedure (modified Bonferroni test) were included in the tables and figures in order to avoid erroneous inferences in tests involving multiple comparisons of outcomes [58, 59].

## Results

### Results at baseline

Before the intervention period, the groups supplemented with synthetic vitamin E (all-rac-α-tocopherol), natural vitamin E (RRR-α-tocopherol) or placebo and the non-intervention group were homogenous with regard to *miR-9-1* and *miR-9-3* methylation levels, anthropometric variables, family income, physical activity practice, menopausal status (Table 1), glycaemic control variables (fasting plasma glucose, haemoglobin A1C, insulin) and serum vitamin E (Table 2).

**Table 1:** Baseline characteristics of the study population before the start of the study^1^

	Synthetic VE (*n* = 12)	Natural VE (*n* = 13)	Placebo (*n* = 9)	Non-intervention (*n* = 10)	*P*-value	*Adjusted p-value* ^*^
DNA methylation (%)						
*miR-9-1*	8.7 ± 8.8	9.3 ± 10.6	12.8 ± 9.0	10.5 ± 9.2	0.637	1.000
*miR-9-3*	8.6 ± 7.7	2.8 ± 4.1	4.0 ± 4.1	5.7 ± 2.6	0.051	0.204
Age (years)	42.1 ± 8.5	49.3 ± 7.7	46.7 ± 11.9	40.1 ± 6.7	0.062	0.248
Body mass index (kg/m^2^)	29.9 ± 4.6	30.4 ± 3.8	30.0 ± 4.5	29.9 ± 3.6	0.918	1.000
Waist circumference (cm)	90.9 ± 10.8	93.0 ± 7.4	92.3 ± 10.7	96.7 ± 7.7	0.518	1.000
Waist-to-height ratio (cm)	0.6 ± 0.1	0.6 ± 0.1	0.6 ± 0.1	0.6 ± 0.1	0.795	1.000
Family income ($)	862.6 ± 317.2	1028.3 ± 652.0	1092.1 ± 1171.1	563.2 ± 447.6	0.155	0.620
Daily physical activity practice/min	10.8 ± 25.4	23.1 ± 32.8	22.2 ± 27.3	10.0 ± 21.1	0.486	1.000
Proportion of active individuals *n* (%)^a^	2(16.7)	4(30.8)	2(22.2)	2(20.0)	0.856	1.000
Proportion Peri and postmenopausal *n* (%)^b^	5(41.7)	9(69.2)	6(66.7)	6(60.0)	0.517	1.000

^1^Data presented as mean and standard deviation or n (%). Synthetic VE contains all-rac-α-tocopherol and natural VE contains natural RRR-α-tocopherol. VE: vitamin E. ^*^Adjusted p-value was calculated by Hommel procedure (modified Bonferroni test). ANOVA and a posteriori Bonferroni test were used to compare means between groups. Kruskal-Wallis test and Mann-Whitney a posteriori test were used to compare medians between groups. Pearson’s chi-square test was used to verify differences between categorical variables: ^a^4 cells (50%) expected counts less than 5; ^b^3 cells (37.5%) expect to count less than 5

**Table 2:** Effects of intervention with α-tocopherol e placebo on glycemic and dietary control variables^1^

	Synthetic VE (*n* = 12)			Natural VE (*n* = 13)			Placebo (*n* = 9)			Analysis of variance (ANOVA) After
	Before	After	*P* ^*^	Before	After	*P* ^*^	Before	After	*P* ^*^	*P* ^*^
HbA_1c_ (%)^2^	5.6 ± 0.3	5.6 ± 0.3	n.s.	5.9 ± 0.3	5.7 ± 0.40	0.004	5.8 ± 0.3	5.8 ± 0.3	n.s.	n.s.
Plasma glucose (mg/dL)^2^	85.4 ± 8.4	92.5 ± 9.4	0.002	94.8 ± 11.3	95.7 ± 10.0	n.s.	94.9 ± 11.8	97.5 ± 11. 1	n.s.	n.s.
Insulin (uU/mL)^2^	12.1 ± 6.8	13.5 ± 6.7	n.s.	12.8 ± 3.4	13.6 ± 5.7	n.s.	14.7 ± 7.6	14.4 ± 5.7	n.s.	n.s.
HOMA-IR^2^	2.6 ± 1.6	3.2 ± 1.8	n.s.	3.0 ± 1.0	3.3 ± 1.7	n.s.	3.6 ± 2.3	3.6 ± 1.8	n.s.	n.s.
Vitamin E (mg/L)^3^	12.2 ± 3.8	23.6 ± 11.2^a^	0.016	12.8 ± 2.6	19.5 ± 7.2	0.002	13.2 ± 6.3	12.3 ± 2.8	n.s.	0.030
α-tocopherol (mg/dia)^4^	0.9 ± 0.8^b^	402.5 ± 2.2^c^	0.004	2.5 ± 3.4	401.4 ± 0.7^c^	0.002	6.6 ± 10.0	2.8 ± 1.4	n.s.	0.000
Calories (kcal/day)^5^	1591.7 ± 358.4	1231.0 ± 263.4	0.006	1340.5 ± 246.8	1049.6 ± 292.8	0.006	1878.1 ± 765.8	1119.9 ± 248.2	0.038	n.s.

^1^Data presented as mean and standard deviation. Synthetic VE contains all-rac-α-tocopherol and contains natural RRR-α-tocopherol. *HbA1c* glycated hemoglobin; *HOMA-IR* Homeostasis Model Assessment-Insulin Resistance; *VE* vitamin E. ^*^Adjusted *p*-value< 0.05 (this p was corrected by Hommel procedure); n.s: not significant. Paired t test or Wilcoxon paired test was used to compare means or medians before and after intervention in each group. ^2^For glycemic control variables no differences were found between the three groups before and after intervention by analysis of variance ANOVA. ^3^For vitamin E (mg/L) differences between the three groups were found only after intervention by ANOVA. Post-Hoc Bonferroni test was used for multiple comparisons after intervention: a = significantly different from placebo group at *p* = 0.004. ^4^For α-tocopherol (mg/day) differences were found before (*p* = 0.037) and after intervention (*p* = 0.000) by the Kruskal-Wallis test. Mann Whitney test was used for multiple comparisons: b = significantly different from placebo group before intervention at *p* = 0.013; c = significantly different from placebo group after intervention at *p* = 0.000. ^5^For calories from diet no differences were found between the three groups before and after intervention by the Kruskal-Wallis test, respectively

The mean BMI in the groups was 30.0 kg/m^2^ (obesity grade 1), with 25% obese women in the synthetic vitamin E group, 46% in the natural vitamin E group, 44% in the placebo group and 30% in the non-intervention group. There were no differences in BMI values among the groups (Table 1).

Regarding the risk classification for diabetes from glycated haemoglobin, 50% of the women in the synthetic vitamin E group, 77% of the women in the natural vitamin E group and 67% in the placebo group presented HbA1c values between 5.7–6.4% [60], without significant differences among the groups before the intervention (Table 3). Serum vitamin E values (mg/L) at the beginning of the intervention were below those recommended by the DRIs related to health effects (≥30 μmol/L).

### Effect of α-tocopherol on DNA methylation

**Fig. 2:**
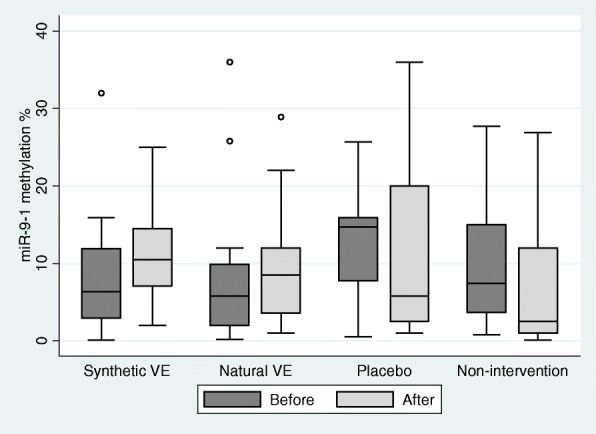
Effect of α-tocopherol supplementation on *miR-9-1* methylation levels. VE: Vitamin E. The synthetic VE contains all-rac-α-tocopherol and contains natural RRR-α-tocopherol. There were no differences in methylation levels before and after intervention for the synthetic vitamin E group (*p* = 0.146; adjusted *p* = 0.584), natural (*p* = 0.581; adjusted *p* = 1.000), placebo (*p* = 1.000; adjusted *p* = 1.000), and non-intervention group (*p* = 0.754; adjusted *p* = 1.000) (Wilcoxon test paired). Adjusted p means that the *p*-value was corrected by Hommel procedure (modified Bonferroni test)

**Fig. 3:**
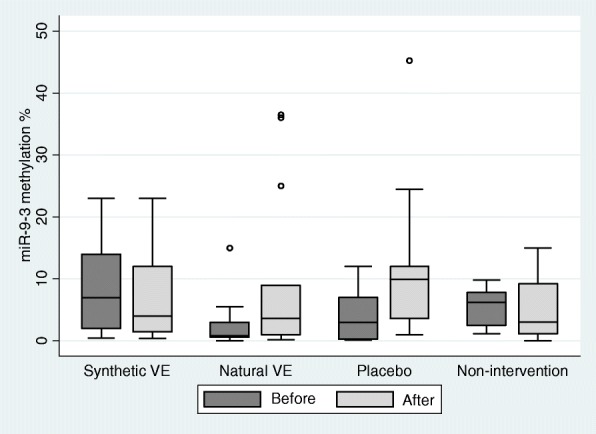
Effect of α-tocopherol supplementation on *miR-9-3* methylation levels. VE: Vitamin E. The synthetic VE contains all-rac-α-tocopherol and contains natural RRR-α-tocopherol. Methylation levels were significantly higher in the natural vitamin E (VE) group after intervention (*p* = 0.000; adjusted *p* = 0.000). There were no differences in methylation levels before and after intervention for the synthetic vitamin E group (*p* = 0.388; adjusted *p* = 0.776), placebo (*p* = 0.179; adjusted *p* = 0.537) and non-intervention group (*p* = 1.000; adjusted *p* = 1.000) (paired Wilcoxon test). Adjusted p means that the p-value was corrected by Hommel procedure (modified Bonferroni test)

*MiR-9-1* methylation levels were unaffected by α-tocopherol (Fig. 2). On the other hand, an elevation in *miR-9-3* methylation levels was observed for the group supplemented with natural source α-tocopherol (Fig. 3). For the placebo group, which did not receive supplementation with α-tocopherol, there was a trend of elevation in miR-9-3 methylation levels; however, this was not significant (Fig. 3).

In order to reduce the variations observed in Fig. 3 we apply the tests with the withdrawal of outliers values. The results did not change (Please see Additional file 1).

### Effects of intervention on biochemical and dietary parameters

The group supplemented with synthetic vitamin E showed an increase in fasting plasma glucose, whereas the group supplemented with natural source vitamin E was the only one to present an HbA1c reduction compared with the baseline (at baseline 77% of the women presented HbA1c values between 5.7–6.4%). Both groups supplemented with vitamin E increased serum levels after intervention. Comparing the three groups, serum levels were higher for the synthetic vitamin E group than for the placebo group. In relation to the α-tocopherol dietary intake levels (mg/day), these were lower than EAR before intervention, being increased only for the groups supplemented with vitamin E. The three groups showed a reduction in caloric intake after intervention (Table 2). There is no difference for HbA1c, plasma glucose, insulin and HOMA-IR after intervention by analysis of variance ANOVA (Table 2).

## Discussion

This study demonstrated that 8 weeks of daily supplementation with natural source vitamin E capsules (RRR-α-tocopherol) in overweight and obese women provided an increase in *miR-9-3* methylation levels and decreased values of glycated haemoglobin. RRR-α-tocopherol is the most biologically active form of α-tocopherol, present in natural sources such as vegetable oils, seeds and nuts. On the other hand, the synthetic form, all-rac-α-tocopherol, is composed of 8 stereoisomers in equal proportions (RRR-, RRS-, RSR-, RSS, SRR-, SRS-, SSR- and SSS-) and consumed as a supplement [26, 30, 44, 61]. Evidence suggests variation of bioavailability and biopotency among of the 8 stereoisomers depending on the dosage, the type of tissue, and the duration of dosing [29, 30]. It has been assumed that, at doses of an equivalent mass, all-rac-α-tocopherol has one-half the biopotency of RRR-α-tocopherol. RRR α**-**tocopherol is preferentially taken up into tissues over and it is suggested better functional capacity [29].

Although all forms of vitamin E are absorbed without preference, the α-tocopherol carrier protein (α-TTP) preferably incorporates RRR-α-tocopherol in VLDL (very low-density lipoprotein) compared to the other stereoisomers. This incorporation into VLDL in the liver is what determines α-tocopherol plasma concentrations [30]. All-rac-α-tocopherol supplement may increase non-RRR-α-tocopherol stereoisomer proportion and consequently decrease RRR-α-tocopherol percentage in plasma [62]. In the present study, α-tocopherol serum levels do not discriminate between the different forms [55]. Thus, we have not been able to evaluate the levels of different stereoisomers in plasma. Different stereoisomers may present different biopotency, which is higher for RRR-α-tocopherol. Therefore, despite the similarity in plasma, other factors such as biopotency could explain the differences between the two sources (synthetic and natural) in relation to the molecular effects. After intervention we found that α-tocopherol serum levels were higher for the synthetic vitamin E group (all-rac-α-tocopherol) comparing to placebo group. We didn’t evaluate lipids markers, but higher α-tocopherol plasma concentrations could also reflect increase plasma lipid concentrations as well as depletion of tissue α-tocopherol [28].

As an important nutrient against oxidative stress, vitamin E is not restricted to antioxidant functions [63]; it also performs several essential actions at the molecular level, such as modulate the activity of nuclear receptors, transcription factors, membrane channels, and enzymes [19]. It is known that epigenetic marks can be altered by a variety of factors [7], including diet [64]. Even though few studies have been conducted regarding the effect of vitamin E on epigenetic mechanisms, in mice, tocopherols were capable of decreasing DNA methylation in different genes [20, 23].

In the present study, we observed increased methylation levels with vitamin E supplementation. However, this was observed only for the *miR-9-3* promoter region and for the group that consumed RRR-α-tocopherol capsules. Taken together, the cited articles and the present study show that vitamin E is associated with changes in the DNA methylation profile and that the decrease or increase in methylation levels is gene-specific. To the best of our knowledge, this is the first study to report the effect of a nutritional intervention on the methylation levels of genes encoding miRs. *MiR-9-3* appears to be the most expressed form among the three genes that are transcribed in miR-9 [14, 65]. Adequate levels of miR-9 are important for the balance of insulin secretion and glucose homeostasis [66]. On the other hand, when miR-9 is overexpressed, *Onecut2* (OC2) expression is reduced, leading to an increase in Granuphilin, a negative regulator of the β-cell secretory ability, resulting in lower insulin secretion. Decrease in the β-cell secretory capacity in turn can cause hyperglycaemia and lead to diabetes [41, 66, 67].

Corroborating these observations, Motawae et al. reported that increased miR-9 expression in human serum was positively associated with fasting glucose and BMI in subjects with type 2 diabetes [15]. These authors did not evaluate methylation levels in genes encoding miR-9. Thus, it is possible that *miR-9-3* promoter region methylation is one of the mechanisms involved in the regulation of miR-9 levels. According to Yan et al., increases in *miR-9-3* promoter region methylation but not *miR-9-1* promoter region methylation were related to the reduction in miR-9 expression in obese rat hepatocytes [14]. In turn, miR-9 may present alternative and non-overlapping functions in different cell types [14, 17, 67].

Regarding the impact on glycaemic control variables, it has been reported that vitamin E would have an effect on glycated haemoglobin and insulin through the disruption of glycation and protection of β-cell toxicity in patients with type 2 diabetes [43, 45] who present with glycated haemoglobin levels of 8–10% or low levels of vitamin E (< 5.0 μg/mL) [45]. A wide variation in the vitamin E form (RRR-α-tocopherol, all-rac-α-tocopherol and tocotrienol) and dosage used (90 to 810 mg/day) have been described in the above-mentioned meta-analysis [43]. In the present study, 77% of obese and overweight women who received 400 mg of RRR-α-tocopherol were in the pre-diabetes group (5.7–6.4 HbA1c%) [60] and presented a significant reduction in this parameter after intervention. Greater benefits on glycaemic control may be observed in treatments with longer periods [43].

Vitamin E supplementation does not appear to result in significant differences for fasting plasma glucose status in individuals with type 2 diabetes compared to placebo or control [43, 45]. In the present study, natural source vitamin E also had no effect on fasting plasma glucose; however, in the all-rac-α-tocopherol group there was an increase in glucose after intervention, despite a significant reduction in the values of the anthropometric markers BMI, waist circumference and waist-to-height ratio and a reduction in dietary caloric intake. As glucose level fell in the normal range and the increase was small, the slight increase did not present any clinical significance. Thus, since indiscriminate vitamin E supplementation is not supported by the available evidence, further efforts are needed to establish biomarkers and selection criteria to predict who can benefit from vitamin E supplementation [26].

Our study has limitations that should be considered in interpreting the results. First, the sample size. Second, we did not measure of miR-9 expression levels and not all CpG sites will present the typical silencing response [20, 68]. However, it’s important to mention there is a possible inverse relationship between *miR-9-3* methylation levels and miR-9 expression level [14]. Significant amounts of miRs have been found not only at the intracellular level but also in human extracellular body fluids [11]. In obesity, circulating miRs have been suggested as biomarkers for the detection of metabolic stress early phases, as well as for the detection of the effects of dietary interventions [9].

## Conclusions

This study randomized, double-blind, exploratory, controlled demonstrated that supplementation with natural source α-tocopherol increased *miR-9-3* promoter region methylation levels and improved glycated haemoglobin profiles in obese and overweight women under energy-restricted diet and NCEP/AHA dietary regimen. These results provide new information on the influence of vitamin E on DNA methylation, specifically on genes encoding miRs.

## Additional file

Additional file 1: Tests with the withdrawal of outliers values. (DOCX 22 kb)

## Abbreviations

24HR: 24-h dietary recalls
BMI: Body mass index
CpG: Cytosine-phosphate-guanine
DRIs: Dietary Reference Intakes
EAR: Estimated Average Requirement
EER: Estimated Energy Requirement
HbA1c: Hemoglobin A1C
HOMA-IR: Homeostasis Model Assessment-Insulin Resistance
miRs: microRNAs
mRNA: messenger RNA
VE: Vitamin E
WC: Waist circumference
WHtR: Waist-to-height ratio

## References

[1] WHO. Obesity and overweight. http://www.who.int/mediacentre/factsheets/fs311/en/. Accessed 9 July 2018.

[2] Bell CG (2017). The Epigenomic analysis of human obesity. Obesity.

[3] Del Carmen Martínez-Jiménez V, Méndez-Mancilla A, Patricia Portales-Pérez D (2018). miRNAs in nutrition, obesity, and cancer: The biology of miRNAs in metabolic disorders and its relationship with cancer development. Mol Nutr Food Res.

[4] Lopomo A, Burgio E, Migliore L (2016). Epigenetics of obesity. Prog Mol Biol Transl Sci.

[5] Wang S, Wu W, Claret FX (2017). Mutual regulation of microRNAs and DNA methylation in human cancers. Epigenetics.

[6] Zhang P, Chu T, Dedousis N, Mantell BS, Sipula I, Li L (2017). DNA methylation alters transcriptional rates of differentially expressed genes and contributes to pathophysiology in mice fed a high fat diet. Mol Metab.

[7] Schübeler D (2015). Function and information content of DNA methylation. Nature.

[8] O’Connell TM, Markunas CA (2016). DNA Methylation and MicroRNA-based biomarkers for risk of type 2 diabetes. Curr Diabetes Rev.

[9] Rome S (2015). Use of miRNAs in biofluids as biomarkers in dietary and lifestyle intervention studies. Genes Nutr.

[10] Fu L, Shi J, Liu A, Zhou L, Jiang M, Fu H (2017). A minicircuitry of microRNA-9-1 and RUNX1-RUNX1T1 contributes to leukemogenesis in t(8;21) acute myeloid leukemia. Int J Cancer.

[11] Cui J, Zhou B, Ross SA, Zempleni J (2017). Nutrition, microRNAs, and human health. Adv Nutr An Int Rev J..

[12] Poddar S, Kesharwani D, Datta M (2017). Interplay between the miRNome and the epigenetic machinery: implications in health and disease. J Cell Physiol.

[13] Padrão NA, Monteiro-Reis S, Torres-Ferreira J, Antunes L, Leça L, Montezuma D (2017). MicroRNA promoter methylation: a new tool for accurate detection of urothelial carcinoma. Br J Cancer.

[14] Yan C, Chen J, Li M, Xuan W, Su D, You H (2016). A decrease in hepatic microRNA-9 expression impairs gluconeogenesis by targeting FOXO1 in obese mice. Diabetologia.

[15] Motawae TM, Ismail MF, Shabayek MI, Seleem MM (2015). MicroRNAs 9 and 370 association with biochemical markers in T2D and CAD complication of T2D. PLoS One.

[16] Chakraborty C, Doss CGP, Bandyopadhyay S, Agoramoorthy G (2014). Influence of miRNA in insulin signaling pathway and insulin resistance: micro-molecules with a major role in type-2 diabetes. Wiley Interdiscip Rev RNA.

[17] Ao R, Wang Y, Tong J, Wang B-F (2016). Altered microRNA-9 expression level is directly correlated with pathogenesis of nonalcoholic fatty liver disease by targeting Onecut2 and SIRT1. Med Sci Monit.

[18] Rashidi B, Hoseini Z, Sahebkar A, Mirzaei H (2017). Anti-atherosclerotic effects of vitamins D and E in suppression of Atherogenesis. J Cell Physiol.

[19] Zingg J-M, Vitamin E, Role in Signal Transduction A (2015). Annu Rev Nutr.

[20] Remely M, Ferk F, Sterneder S, Setayesh T, Kepcija T, Roth S (2017). Vitamin E modifies high-fat diet-induced increase of DNA strand breaks, and changes in expression and DNA methylation of Dnmt1 and MLH1 in C57BL/6J male mice. Nutrients.

[21] Gaedicke S, Zhang X, Schmelzer C, Lou Y, Doering F, Frank J (2008). Vitamin E dependent microRNA regulation in rat liver. FEBS Lett.

[22] de la Iglesia R, Mansego ML, Sánchez-Muniz FJ, Zulet MA, Martinez JA (2014). Arylesterase activity is associated with antioxidant intake and paraoxonase-1 (PON1) gene methylation in metabolic syndrome patients following an energy restricted diet. EXCLI J.

[23] Huang Y, Shu L, Saw CL-L, Wu T-Y, Suh N, Khor TO (2012). A γ-tocopherol-rich mixture of tocopherols maintains Nrf2 expression in prostate tumors of TRAMP mice via epigenetic inhibition of CpG methylation. J Nutr.

[24] Mah E, Sapper TN, Chitchumroonchokchai C, Failla ML, Schill KE, Clinton SK (2015). α-Tocopherol bioavailability is lower in adults with metabolic syndrome regardless of dairy fat co-ingestion: a randomized, double-blind, crossover trial. Am J Clin Nutr.

[25] Traber MG, Mah E, Leonard SW, Bobe G, Bruno RS (2017). Metabolic syndrome increases dietary alpha-tocopherol requirements as assessed using urinary and plasma vitamin E catabolites: a double-blind, crossover clinical trial. Am J Clin Nutr.

[26] Galli F, Azzi A, Birringer M, Cook-Mills JM, Eggersdorfer M, Frank J (2017). Vitamin E: emerging aspects and new directions. Free Radic Biol Med.

[27] Péter S, Friedel A, Roos FF, Wyss A, Eggersdorfer M, Hoffmann K, et al. A systematic review of global alpha-tocopherol status as assessed by nutritional intake levels and blood serum concentrations. Int J Vitam Nutr Res. 2015;85:261–81. 10.1024/0300-9831/a000281 27414419

[28] Traber MG, Vitamin E (2014). Inadequacy in humans: causes and consequences. Adv Nutr An Int Rev J.

[29] Ranard KM, Erdman JW (2018). Effects of dietary RRR α-tocopherol vs all-racemic α-tocopherol on health outcomes. Nutr Rev.

[30] Zhao Y, Monahan FJ, McNulty BA, Brennan L, Gibney MJ, Gibney ER (2015). Tocopherol stereoisomers in human plasma are affected by the level and form of the vitamin E supplement used. J Nutr.

[31] Santolim LV, do MEC A, Fachi JL, Mendes MF, de Oliveira CA (2017). Vitamin E and caloric restriction promote hepatic homeostasis through expression of connexin 26, N-cad, E-cad and cholesterol metabolism genes. J Nutr Biochem.

[32] Toraño EG, García MG, Fernández-Morera JL, Niño-García P, Fernández AF (2016). The impact of external factors on the epigenome: in utero and over lifetime. Biomed Res Int.

[33] de Oliveira Y, RPA L, RCP L, MGA M, da CSO S, do RAF N (2018). Decrease of the DNA methylation levels of the ADRB3 gene in leukocytes is related with serum folate in eutrophic adults. J Transl Med.

[34] Costa MJ de C. Cycle II of Diagnosis and Intervention of the Food, Nutritional and Non-Communicable Diseases Status of the Population of the Municipality of João Pessoa (II DISANDNT/JP). João Pessoa: Foundation for Support and Research Support/PPSUS/PB/Ministry of Health/National Research Council/CNPq/João Pessoa Prefecture. Public notice 001/2013, number EFP_00008187. http://fapesq.rpp.br/editais-resultados/resultadofinalppsus2013.pdf/view. Accessed 9 July 2018.

[35] National Cholesterol Education Program (NCEP) (2002). Expert panel on detection, evaluation and T of HBC in a adult TPI. Third report of the National Cholesterol Education Program (NCEP) expert panel on detection, evaluation, and treatment of high blood cholesterol in adults (adult treatment panel III) final report. Circulation.

[36] Lichtenstein AH, Appel LJ, Brands M, Carnethon M, Daniels S, Franch HA (2006). Diet and lifestyle recommendations revision 2006: a scientific statement from the American heart association nutrition committee. Circulation.

[37] de C CMJ (2013). Nutrição Clínica: Uso do Sistema de Equivalentes na Prática Dietoterápica.

[38] Institute of Medicine F and NB (2002). Dietary reference intakes for energy, carbohydrate, Fiber, fat, fatty acids, cholesterol, protein and amino acids.

[39] Associação Brasileira para o Estudo da Obesidade e da Síndrome Metabólica (Abeso) (2009). Diretrizes Brasileiras de Obesidade 2009-2010.

[40] Bray GA, Frühbeck G, Ryan DH, Wilding JPH (2016). Management of obesity. Lancet.

[41] Ramachandran D, Roy U, Garg S, Ghosh S, Pathak S, Kolthur-Seetharam U (2011). Sirt1 and mir-9 expression is regulated during glucose-stimulated insulin secretion in pancreatic β-islets. FEBS J.

[42] Hoskins A, Roberts JL, Milne G, Choi L, Dworski R (2012). Natural-source d-α-tocopheryl acetate inhibits oxidant stress and modulates atopic asthma in humans in vivo. Allergy.

[43] Xu R, Zhang S, Tao A, Chen G, Zhang M (2014). Influence of vitamin E supplementation on glycaemic control: a meta-analysis of randomised controlled trials. PLoS One.

[44] Otten JJ, Hellwig JP, Linda D (2006). Dietary reference intakes: the essential guide to nutrient.

[45] Suksomboon N, Poolsup N, Sinprasert S (2011). Effects of vitamin E supplementation on glycaemic control in type 2 diabetes: systematic review of randomized controlled trials. J Clin Pharm Ther.

[46] Jansen E, Viezeliene D, Beekhof P, Gremmer E, Ivanov L (2016). Tissue-specific effects of vitamin e supplementation. Int J Mol Sci.

[47] Blumberg J, Bailey R, Sesso H, Ulrich C (2018). The evolving role of multivitamin/multimineral supplement use among adults in the age of personalized nutrition. Nutrients.

[48] World Health Organization (1995). Physical status: the use and interpretation of anthropometry. Report of a WHO expert committee.

[49] Ross R, Berentzen T, Bradshaw AJ, Janssen I, Kahn HS, Katzmarzyk PT (2008). Does the relationship between waist circumference, morbidity and mortality depend on measurement protocol for waist circumference?. Obes Rev.

[50] Ashwell M, Gibson S (2016). Waist-to-height ratio as an indicator of ‘early health risk’: simpler and more predictive than using a ‘matrix’ based on BMI and waist circumference. BMJ Open.

[51] de FEL L, Latorre MDRDDO, Costa MJDC, Fisberg RM (2008). Diet and cancer in Northeast Brazil: evaluation of eating habits and food group consumption in relation to breast cancer. Cad saude publica / Minist da Saude, Fund Oswaldo Cruz, Esc Nac Saude Publica.

[52] Cooper GR (1973). Methods for determining the amount of glucose in blood. CRC Crit Rev Clin Lab Sci.

[53] Khuu HM, Robinson CA, Goolsby K, Hardy RW, Konrad RJ (1999). Evaluation of a fully automated high-performance liquid chromatography assay for hemoglobin A1c. Arch Pathol Lab Med.

[54] Peplies J, Jiménez-Pavón D, Savva SC, Buck C, Günther K, Fraterman A (2014). Percentiles of fasting serum insulin, glucose, HbA1c and HOMA-IR in pre-pubertal normal weight European children from the IDEFICS cohort. Int J Obes.

[55] Karpińska J, Mikołuć B, Motkowski R, Piotrowska-Jastrzębska J (2006). HPLC method for simultaneous determination of retinol, α-tocopherol and coenzyme Q10 in human plasma. J Pharm Biomed Anal.

[56] Miller SA, Dykes DD (1988). Polesky HF. A simple salting out procedure for extracting DNA from human nucleated cells. Nucleic Acids Res.

[57] Tsai KW, Liao YL, Wu CW, Hu LY, Li SC, Chan WC (2011). Aberrant hypermethylation of miR-9 genes in gastric cancer. Epigenetics..

[58] Hommel GA (1988). Stagewise Rejective multiple test procedure based on a modified Bonferroni test. Biometrika.

[59] Wright SP (1992). Adjusted P-values for simultaneous Inferencele. Biometrics.

[60] American Diabetes Association. 2 (2017). Classification and Diagnosis of Diabetes. Diabetes Care..

[61] Borel P, Desmarchelier C (2016). Genetic variations involved in vitamin E status. Int J Mol Sci.

[62] Gaur S, Kuchan MJ, Lai C-S, Jensen SK, Sherry CL (2017). Supplementation with RRR- or all-rac -α-tocopherol differentially affects the α-tocopherol stereoisomer profile in the milk and plasma of lactating women. J Nutr.

[63] Wong M, Lodge JK (2012). A metabolomic investigation of the effects of vitamin E supplementation in humans. Nutr Metab (Lond).

[64] Switzeny OJ, Müllner E, Wagner K-H, Brath H, Aumüller E, Haslberger AG (2012). Vitamin and antioxidant rich diet increases MLH1 promoter DNA methylation in DMT2 subjects. Clin Epigenetics.

[65] Senyuk V, Zhang Y, Liu Y, Ming M, Premanand K, Zhou L (2013). Critical role of miR-9 in myelopoiesis and EVI1-induced leukemogenesis. Proc Natl Acad Sci U S A.

[66] Plaisance V, Abderrahmani A, Perret-Menoud V, Jacquemin P, Lemaigre F, Regazzi R (2006). MicroRNA-9 controls the expression of Granuphilin/Slp4 and the secretory response of insulin-producing cells. J Biol Chem.

[67] Fernandez-Valverde SL, Taft RJ, Mattick JS (2011). MicroRNAs in β-cell biology, insulin resistance, diabetes and its complications. Diabetes.

[68] Carraro JCC, Hermsdorff HHM, Mansego ML, Zulet MÁ, Milagro FI, Bressan J (2016). Higher fruit intake is related to TNF-alpha Hypomethylation and better glucose tolerance in healthy subjects. J Nutrigenet Nutrigenomics.

