# Supplementary material for: α-Tocopherol influences glycaemic control and miR-9-3 DNA methylation in overweight and obese women under an energy-restricted diet: a randomized, double-blind, exploratory, controlled clinical trial
Source: Nutr Metab (Lond). 2018 Jul 11;15:49. doi: 10.1186/s12986-018-0286-7 (PMC6042339; doi:10.1186/s12986-018-0286-7)
Supplement: Supplementary file 1 — Tests with the withdrawal of outliers values. (DOCX 22 kb) [file 12986_2018_286_MOESM1_ESM.docx]

**Additional file 1. Tests with the withdrawal of outliers values**

Removed outliers (individuals with % mir-9-3 methylation above 30)

Test: paired Wilcoxon test

We excluded 3 individuals:
Group VE Synthetic = 12
Group VE Natural = 11 (withdrawn 2 individuals)
Group Placebo = 8 (withdrawn 1 individual)
Group non-intervention = 10

*Plasma glucose (fasting glycemia): RESULTS REMAINED SIGNIFICANT ONLY FOR VE SYNTHETIC GROUP

VE SYNTHETIC

plasma glucose1 = plasma glucose2

z = -3.025

Prob > |z| = 0.0025

VE NATURAL GROUP

Ho: plasma glucose1 = plasma glucose2

z = -0.551

Prob > |z| = 0.5819

PLACEBO

Ho: plasma glucose1 = plasma glucose2

z = -1.400

Prob > |z| = 0.1614

*% mir-9-3 methylation: RESULTS REMAINED SIGNIFICANT ONLY FOR VE NATURAL GROUP

VE SYNTHETIC

Ho: methy_pre_9_3 = methy_pos_9_3

z = 0.000

Prob > |z| = 1.0000

VE NATURAL

Ho: methy_pre_9_3 = methy_pos_9_3

z = -2.934

Prob > |z| = 0.0033

PLACEBO

Ho: methy_pre_9_3 = methy_pos_9_3

z = -1.120

Prob > |z| = 0.2626

NON-INTERVENTION

Ho: methy_pre_9_3 = methy_pos_9_3

z = 0.357

Prob > |z| = 0.7213

*HbA1c: RESULTS REMAINED SIGNIFICANT ONLY FOR VE NATURAL GROUP

VE SYNTHETIC

Ho: hba1c1 = hba1c2

z = -0.199

Prob > |z| = 0.8424

VE NATURAL

Ho: hba1c1 = hba1c2

z = 2.369

Prob > |z| = 0.0179

PLACEBO

Ho: hba1c1 = hba1c2

z = -0.211

Prob > |z| = 0.8328

*% mir-9-1 methylation:

VE SYNTHETIC

Ho: methy_pre_9_1 = methy_pos_9_1

z = -1.334

Prob > |z| = 0.1823

VE NATURAL

Ho: methy_pre_9_1 = methy_pos_9_1

z = -0.889

Prob > |z| = 0.3739

PLACEBO

Ho: methy_pre_9_1 = methy_pos_9_1

z = -0.420

Prob > |z| = 0.6744

NON-INTERVENTION

Ho: methy_pre_9_1 = methy_pos_9_1

z = 0.561

Prob > |z| = 0.5751

Removed outliers (individuals with % mir-9-3 methylation above 25)

Test: paired Wilcoxon test

We excluded 4 individuals:
Group VE Synthetic = 12
Group VE Natural = 10 (withdrawn 3 individuals)
Group Placebo = 8 (withdrawn 1 individual)
Group non-intervention = 10

*HbA1c: RESULTS REMAINED SIGNIFICANT ONLY FOR VE NATURAL GROUP

VE Synthetic

Ho: hba1c1 = hba1c2

z = -0.199

Prob > |z| = 0.8424

VE Natural

Ho: hba1c1 = hba1c2

z = 2.143

Prob > |z| = 0.0321

Placebo

Ho: hba1c1 = hba1c2

z = -0.211

Prob > |z| = 0.8328

*% mir-9-3 methylation: RESULTS REMAINED SIGNIFICANT ONLY FOR VE NATURAL GROUP

VE Synthetic

Ho: methy_pre_9_3 = methy_pos_9_3

z = 0.000

Prob > |z| = 1.0000

VE Natural

Ho: methy_pre_9_3 = methy_pos_9_3

z = -2.803

Prob > |z| = 0.0051

Placebo

Ho: methy_pre_9_3 = methy_pos_9_3

z = -1.120

Prob > |z| = 0.2626

Non-intervention

Ho: methy_pre_9_3 = methy_pos_9_3

z = 0.357

Prob > |z| = 0.7213

*Plasma glucose (fasting glycemia): RESULTS REMAINED SIGNIFICANT ONLY FOR VE SYNTHETIC GROUP

VE SYNTHETIC

Ho: plasma glucose1 = plasma glucose2

z = -3.025

Prob > |z| = 0.0025

VE Natural

Ho: plasma glucose1 = plasma glucose2

z = -0.572

Prob > |z| = 0.5674

Placebo

Ho: plasma glucose1 = plasma glucose2

z = -1.400

Prob > |z| = 0.1614

*% mir-9-1 methylation:

VE SYNTHETIC

Ho: methy_pre_9_1 = methy_pos_9_1

z = -1.334

Prob > |z| = 0.1823

VE Natural

Ho: methy_pre_9_1 = methy_pos_9_1

z = -0.663

Prob > |z| = 0.5076

Placebo

Ho: methy_pre_9_1 = methy_pos_9_1

z = -0.420

Prob > |z| = 0.6744

Non-intervention

Ho: methy_pre_9_1 = methy_pos_9_1

z = 0.561

Prob > |z| = 0.5751
